# Green roofs in the tropics: design considerations and vegetation dynamics

**DOI:** 10.1016/j.heliyon.2020.e04712

**Published:** 2020-08-19

**Authors:** Iana F. Grullón – Penkova, Jess K. Zimmerman, Grizelle González

**Affiliations:** a Department of Environmental Sciences, University of Puerto Rico, 17 Avenida Universidad, Río Piedras, PR 00921, USA; b International Institute of Tropical Forestry, USDA Forest Service, Jardín Botánico Sur, 1201 Calle Ceiba, Río Piedras, PR 00926-1119, USA

**Keywords:** Environmental science, Ecology, Environmental management, Sustainable development, Urbanization, Ecosystem services, Biodiversity, Green roofs, Vegetation dynamics, Green roof ecosystems, Green roof management, Tropical green roofs, Puerto Rico

## Abstract

Green roofs (GR) have been proposed as a possible solution for urban stressors that, integrated with other remediation and mitigation actions, can lead the way to a more sustainable society. Even when some aspects of green roof design are well established and known (i.e. depth arrangements, materials, structural components, etc.) there is a need for further development on ecological attributes. This study is a descriptive analysis of suitable plant species for their possible incorporation in green roof designs with tropical climate conditions. Green roof research has been mostly led by temperate climate countries and has neglected to address tropical areas; this study aims to move research towards this knowledge gap. The evaluation of the vegetation dynamics in these novel ecosystems was done through a case study in the renovated facilities of the International Institute of Tropical Forestry in Río Piedras, Puerto Rico, which incorporated a set of green roofs in their infrastructure. We also sampled an older green roof built in the Social Sciences Faculty at the University of Puerto Rico at Río Piedras. A three-dimensional approach, the Point-Intercept Method, was taken in the vegetation surveys to capture as much as possible the green infrastructure of the roofs. Most of the originally planted species did not appear in these surveys. On the contrary, mainly new species dominated the areas. Along with the findings of these surveys and those in other tropical countries, a list of suitable species for green roofs in Puerto Rico is suggested, and some general recommendations are made for the better management of green roofs in tropical zones.

## 1 Introduction

Rapid economic growth of countries and the accelerated urban increase, along with multiple problems associated with urban sites, like air, water, and soil quality deterioration, vegetation loss, different source contamination hotspots, among others, have created the necessity for the implementation of new solutions to the challenges of urban living (Berardi et al., 2014). Urban expansion at the expense of green areas translates into a reduction in canopy interception which causes temperature increase and air humidity decrease (Vijayaraghavan, 2016), alteration of city heat balances, among other problems. Green roofs (GR) are presented as one of the multiple mitigation tools that can be incorporated into city plans to offset climate change effects, urban expansion problems, and other possible concerns related to human intervened ecosystems. GR are novel ecosystems created through the intervention of ecological inputs in the design process in which many considerations are made for several key aspects for better performance and achievement of desire outcomes. In that sense, what some experts suggest is to build taking in consideration functional diversity (FD), in the belief that greater biodiversity would translate into ecosystem stability (Van Mechelen et al., 2015, Van Mechelen et al., 2015b). Authors highlight that a careful selection of the pool of species would translate in an increase in stability, outcomes, durability and resilience of the ecosystem (Berardi et al., 2014; Van Mechelen et al., 2015; Van Mechelen et al., 2015, Van Mechelen et al., 2015b). Another important aspect is soil depth, in most of the cases the substrate is made of a mixture of different proportions of compost with: crushed bricks, expanded clay, and/or clay-loam soil; in addition the mixture may contain animal manure and green wastes such as plant pruning and debris to increase nutrients availability (Ondoño et al., 2015). The soil depth is relevant for multiple reasons: the type of plants it can support, the amount of insulation it can provide to the building in terms of external heat, sound isolation, water filtration, among many other aspects (Stovin et al., 2015; Gargari et al., 2016).

GR are classified by two main attributes, substrate depth and/or installment method. For the first classification GR are divided in extensive (below 200 mm) and intensive (above 200 mm). Extensive green roofs are shallower in depth and require less maintenance. Intensive green roofs are more expensive, provide mostly accessible areas with recreational purposes, have heavier weight and high plant diversity (Berardi et al., 2014). For instance, Berardi et al. (2014) state that “the types of plants that can be utilized for extensive green roofs are limited, and both the energy performance and storm water management potentials are relatively low”. This problem arises from the resource availability of the substrate, the community interaction of available species, and the low maintenance provided to these systems; which translates into a reduction of outcomes from the services desired from GR (i.e., energy and heat balance regulation, storm water reduction and filtration, biodiversity enhancement, etc.). The implementation clasification, on the other hand, is based on the procedure of construction or design and the categories are: pre-cultivated, modular, or layered type, depending on simplicity, time, and the type of system and cost of the building process (Berardi et al., 2014).

As GR have become more popular in the recent decades and their implementation has expanded beyond Europe, the urge to understand how this novel ecosystem function has arisen in many areas. Various studies have been conducted to assess the novelty and suitability of green roofs in non-tropical areas, but very few have evaluated performance of green roofs in tropical settings (Lugo and Rullán, 2015). The tropical region contains a great amount of the world biodiversity and counts with a different climatic condition than that on the temperate zone. For this reason, a careful evaluation needs to be done to measure how to adapt the design to the climatic features of tropical GR. Puerto Rico is an island located in the Caribbean Region, with intense urbanization problems and widely spread urban areas. As a territory of the United States of America, many standards and policies in the Island as written for the mainland USA also apply to Puerto Rico (Rudel et al., 2000). Puerto Rico has started to implement, from individually stimulated efforts, GR technology. Researchers have found that, if approached from a public policy level, the popularity and therefore the cost of sustainability initiatives such as green roofs could be improved and become accessible not only for the organizational level but for the individual as well (Peng Lihua, 2012; Sihau, 2008; Wong et al., 2003). Moreover, in terms of governance, public policies play a quality control roll in terms of efficiency and effectiveness both by allocating resources and securing adaptation action (Mees et al., 2012). Besides incentives and subsidies, another approach is to include green roof standards and codes in city building ordinances; places like Tokyo (Japan), Linz (Austria), Basel (Switzerland), Toronto (Canada), as well as some US cities like Portland (Oregon), and Chicago (Illinois) have dictated specific guidelines for incrementing green roof coverage by incorporation in new designs or reconstructions (Carter and Fowler, 2008; Oberndorfer et al., 2007, Oberndorfer et al., 2007; Olsen, 2015). A comprehensive list of GRs in the island is lacking, but buildings such as the *Cuartel de Ballajá*, Music Conservatory, *Banco Popular* Tower, the International Institute of Tropical Forestry, and the Social Sciences Faculty of the UPR-RP, among other sites, have already installed green roofs in Puerto Rico as in the rest of the tropics, the green roof systems have been recently explored and therefore not deeply studied for its broad implementation. In contrast with countries which have taken a public policy approach towards this issue, Puerto Rico has acted sporadically and in individual and small attempts in the implementation of green roofs. The research performed to evaluate the performance and adaptability of the green roofs in Puerto Rico are scarce and ultimately address the benefits to be obtained by said structure rather that its accommodation to the local conditions. This study aims to address some of the questions that arise from the design adaptability in the tropical settings, by answering the following:

1. How does originally planted species list and current surveyed species compare?
2. What were the most dense and frequent species among green roof depths?
3. What set of species is more suitable for their incorporation on green roof design in tropical environmental conditions?

## 2 Methods

### 2.1 Study site

**Figure 1:**
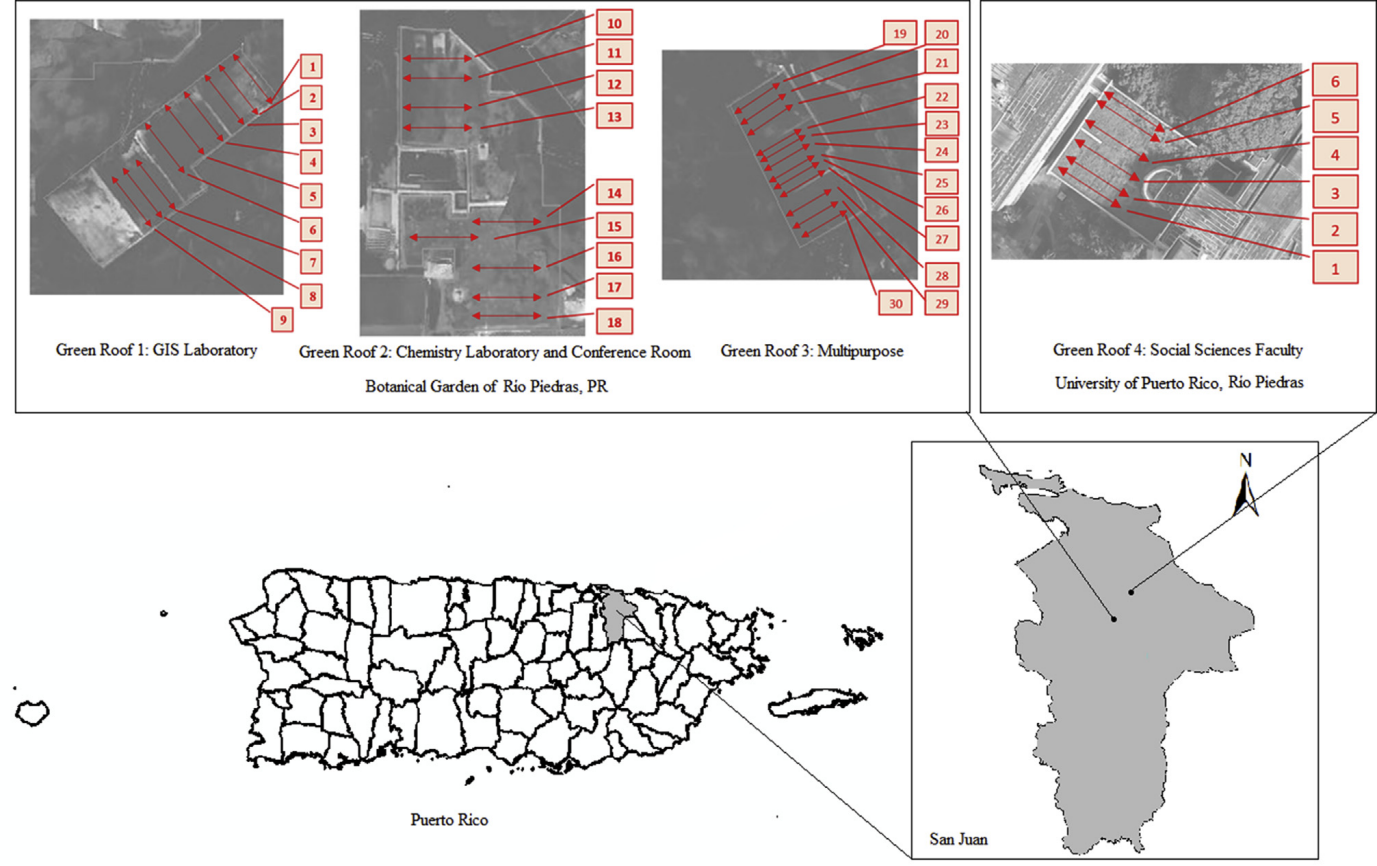
Sampled transects on the green roofs at the Botanical Garden in Río Piedras (GR 1,2,3) and the University of Puerto Rico, Río Piedras Campus, PR (GR 4).

The case study of tropical green roof vegetation dynamics was completed at the renovated facilities of the International Institute of Tropical Forestry on the campus of the University of Puerto Rico in Río Piedras, Puerto Rico. The Institute, located in the Botanical Garden of the University of Puerto Rico has five buildings, green roofs were installed on four of them: the GIS and Remote Sensing Laboratory, Chemistry Laboratory Annex, Technology Transfer Conference Center, and a Multipurpose Building (Figure 1). Employees of the Institute formally inaugurated their GR on May 22nd, 2013, by the time of sampling the roofs had been installed for 4 years.

The GR installed at the Institute facilities are both extensive and intensive in design. The layout and design of the GR was intended for experimentation on the benefits of green roofs. The GIS and Multipurpose buildings were sub-divided into seven (7) separate green roofs and one (1) cool roof. Soil depths were varied in each GR sub-division (5.08cm, 7.62cm, 10.16cm, 12.7cm, 15.24cm, 20.32cm, & 25.4cm) (Table 1). The remaining buildings did not incorporate this division scheme. All green roofs were previous cool roofs as described by Lugo and Rullán (2015). In 2012 a total of 26 species were originally planted (around 16,000 plugs were installed on the project, with some seeds) (Table 2).

**Table 1:** Green Roofs identification and description.

Building Location	Building Purpose	Roof Number	Green Roof Depths	Transects Numbers
Botanical Garden of Río Piedras, P.R.	GIS Laboratory	1	cool roof 5.08 cm 7.62 cm 10.16 cm	--- 7–9 4–6 1–3
Botanical Garden of Río Piedras P.R.	Chemistry Laboratory & Conference Room	2	10.16 cm	10–18
Botanical Garden of Río Piedras, P.R.	Multipurpose	3	12.7 cm 15.24 cm 20.32 cm 25.4 cm	28–30 25–27 22–24 19–21
University of Puerto Rico, Río Piedras	Social Sciences Faculty	4	N/A^1^	31–36

1 N/A: Not available Information.

**Table 2:** List of species originally planted (OP) and spontaneous vegetation (SV) at the green roofs of the International Institute of Tropical Forestry classified on dominance (C, common and R, rare).

Species name	Species code	Origin	Growth Form	Rarity
*Agapanthus praecox*	AGA PRA	OP	Herb	R
*Aloe barbadensis*	ALO VER	OP	Succulent	R
*Alopecurus pratensis*	FOX GRA	SV	Grass	R
*Aptenia cordifolia*	APT COR	OP	Succulent	R
*Arachis hypogaea*	ARA HYP	OP/SV	Herb	R
*Asclepias curassavica*	ASC CUR	SV	Herb	R
*Bidens alba*	BID ALB	SV	Herb	C
*Bulbine caulescens*	BUL CAU	SV	Succulent	R
*Capobrotus edulis*	CAP EDU	OP	Herb	R
*Cissus verticillata*	CIS VER	SV	Vine	R
*Crassula muscosa*	CRA MUS	OP	Succulent	R
*Cymbopogon ambiguus*	CYM AMB	OP/SV	Grass	R
*Cyperaceae kyllinga*	CYP KYL	SV	Sedges	C
*Delosperma sutherlandii*	DEL SUT	OP	Succulent	R
*Desmodium spp*	DES SPP	SV	Herb	R
*Diodia spp*	DIO SPP	SV	Herb	R
*Emilia fosbergii*	EMI FOS	SV	Herb	R
*Emilia sonchifolia*	EMI SON	SV	Herb	R
*Euphorbia graminea*	EUP GRA	SV	Herb	R
*Ipomea spp*	IPO SPP	SV	Vine	R
*Kalanchoe pinnata*	KAL PIN	SV	Succulent	R
*Kalanchoes x houghtonii*	KAL X HOU	SV	Succulent	R
*Lampranthus deltoides*	LAM DEL	OP	Succulent	R
*Macroptilium lathyroides*	MAC LAT	SV	Herb	R
*Malephora crocea*	MAL CRO	OP	Succulent	R
*Malephora lutea*	MAL LUT	OP	Herb	R
*Melothria pendula*	MEL PEN	SV	Vine	R
*Momordica charantia*	MOM CHA	SV	Vine	C
*Nephrolepis multiflora*	NEP MUL	SV	Fern	R
*Oxalis articulata*	OXA ART	SV	Herb	R
*Oxalis corniculata*	OXA COR	SV	Herb	R
*Passiflora foetida*	PAS FOE	OP/SV	Vine	R
*Paspalum paniculatum*	PAS GRA	SV	Grass	C
*Penstemon pinifolius*	PEN PIN	OP	Herb	R
*Portulacaria afra*	POR AFR	SV	Succulent	R
*Portulaca grandiflora*	POR GRA	SV	Herb	R
*Portulaca oleracea*	POR OLE	SV	Herb	R
*Portulaca pilosa*	POR PIL	SV	Herb	R
*Stachytarpheta jamaicensis*	PUR FLO	SV	Shrub	R
*Rhoeo spathacea*	RHO SPA	OP	Shrub	R
*Rosmarinus officinalis*	ROS OFF	OP	Shrub	R
*Ruschia pulvinaris*	RUS PUL	OP	Succulent	R
*Sansevieria cylindrica*	SAN CYL	OP	Succulent	R
*Sansevieria hahnii*	SAN HAH	OP	Succulent	R
*Sedum dasyphyllum*	SED DAS	OP	Succulent	R
*Sedum mexicanum*	SED MEX	OP	Succulent	R
*Sedum pulchellum*	SED PUL	OP	Succulent	R
*Sedum rubrotinctum*	SED RUB	OP	Succulent	R
*Sedum Stahlii*	SED STA	OP/SV	Succulent	R
*Spermacoce verticillata*	SPE VER	SV	Shrub	R
*Spigelia anthelmia*	SPI ANT	SV	Herb	R
*Stapelia grandiflora*	STA GRAN	OP	Succulent	R
*Talinum paniculatum*	TAL PAN	OP/SV	Herb	R
*Thunbergia spp*	THU SPP	SV	Vine	C
*Tulbaghia violacea*	TUL VIO	OP/SV	Herb	R
*Unknown species # 1*	UNK 4-1	SV	---	R
*Unknown species # 2*	UNK 4-2	SV	---	R

**Figure 2:**
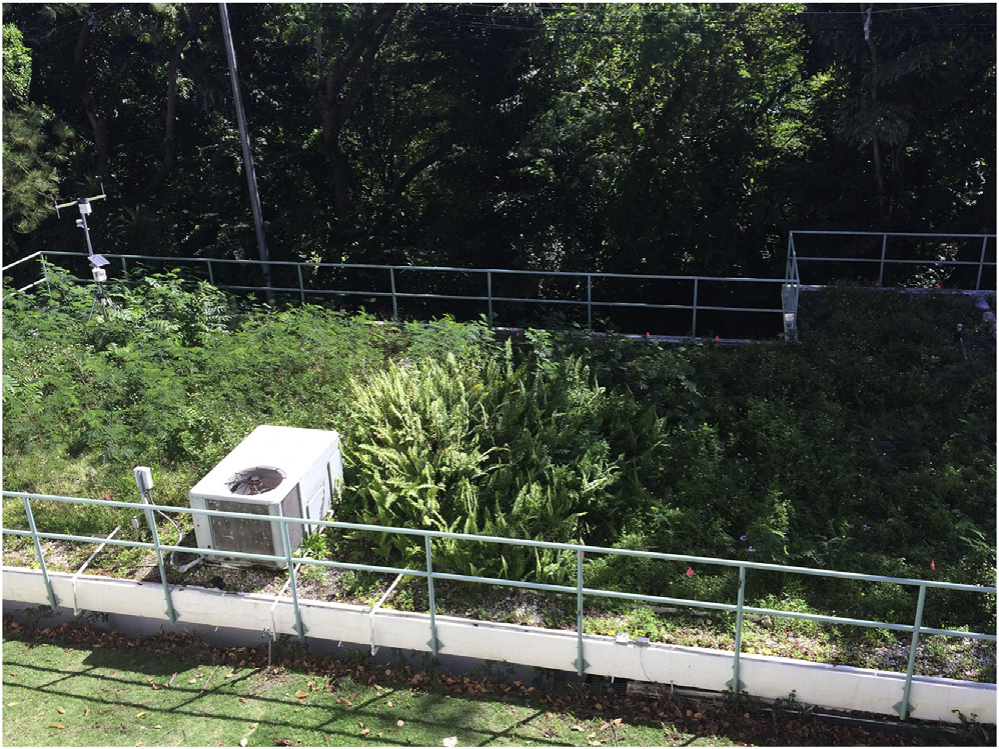
GIS Laboratory (Green Roof # 0–3) years after installment with no maintenance (Image by Author).

Maintenance has been minimum throughout the years, limited to a first-year periodical weeding without irrigation or fertilization (Lugo and Rullán, 2015). The lack of maintenance has allowed spontaneous vegetation to colonize the areas (Figure 2) and originally planted species have either diminished in coverage or disappeared; occasional tree growth can be seen as well. An additional green roof on the UPR – Río Piedras campus was also incorporated into this case study. This one stands above the Social Sciences Faculty's building and is more than 20 years old. The design and structure differ from modern green roofs but since it does not receive any maintenance either it serves as a good platform for spontaneous vegetation studies. Dr. Carlos Severino (*personal communication*) informed us that at the beginning there were only two species involved in the design, *Kalanchoe tubiflora* and *Kalanchoe daigremontiana*.

### 2.2 Vegetation surveys

All roofs were sampled along 1 m transects from north to south using a random number generator to locate them. The ends of transects were permanently marked for future surveys and a long-term evaluation of the vegetation of the green roofs. To determine the abundance of herbaceous species on green roofs, we used the “Point-Intercept Method” as described by Mueller-Dombois and Ellenberg (1974). This method samples the three-dimensional layout of plant structure by counting the number of “touches” by pins lowered through the vegetation every 20 cm. It is a nondestructive measure of plant abundance that avoids the subjectivity of visual cover estimates. A 1m frame with ten pins was placed every 20 cm along 3 different transects (per depth) placed randomly along each roof; for GR # 2 & 4 since there was no variation in the substrate depths, 9 and 6 transects were survey respectively. Transects were 7 m long on the GR # 1, 2, and 3. Because of roof size and shape GR # 4 at the Social Sciences Faculty the length was increased to 14 m. Counts of touches were aggregated by 1 × 1 m quadrats.

The abundance of each species was summarized by the sum of touches per species over all touches (relative density) and presence vs. absence in 1 m^2^ quadrats over the sum of the number of quadrats (relative frequency). These values helped distinguish the most abundant versus widespread (but sparsely vegetated) species. Also, an Importance Value (IV) was calculated for each species, by summing relative density (%) and relative frequency (%) to obtain a 0–200 value. IV is a commonly used index because it comprises both presence and abundance (Curtis and McIntosh, 1951; Dai et al., 2018). Relationship between variables was evaluated and graphed through R Statistical Software (R Core Team, 2016).

## 3 Results

**Figure 3:**
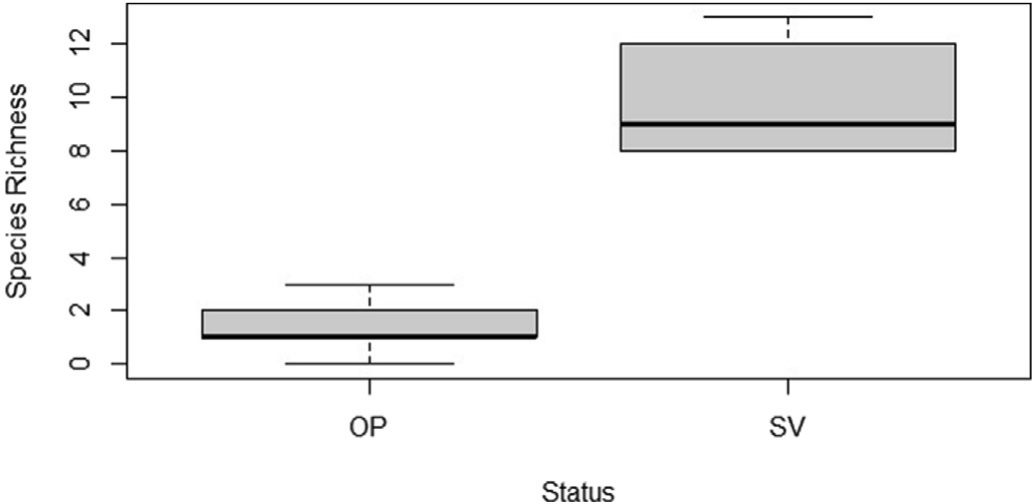
Comparison of species richness per origin status of species over all roofs. OP: Originally planted, SV: Spontaneous vegetation.

**Figure 4:**
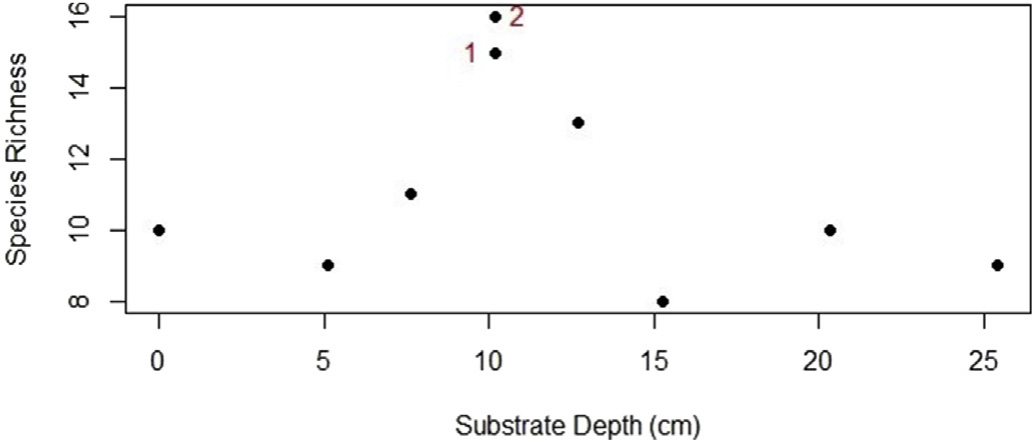
Species richness per substrate depth of green roofs at the International Institute of Tropical Forestry. Green roofs # 1 and 2 (marked in red) were the ones with the highest species richness, both with a 10.16 cm substrate depth.

Only six species survived from the original 26 planted species; those were: *Arachis hypogaea*, *Cymbopogon ambiguus*, *Passiflora foetida*, *Sedum stahlii*, *Talinum paniculatum*, *Tulbaghia violacea*. Thus, species richness was greater for plants of spontaneous origin, in contrast with the originally planted ones, regardless of the depth (Figure 3). When substrate depth was evaluated in terms of species richness, GR # 1 & 2 at 10.16 cm exhibited the most diverse communities, with a total of 15 and 16 different species respectively (Figure 4). For both roofs at this depth the proportion of originally planted to spontaneous vegetation was 2–13 (GR # 1), and 3 to 13 (GR # 2).

**Figure 5:**
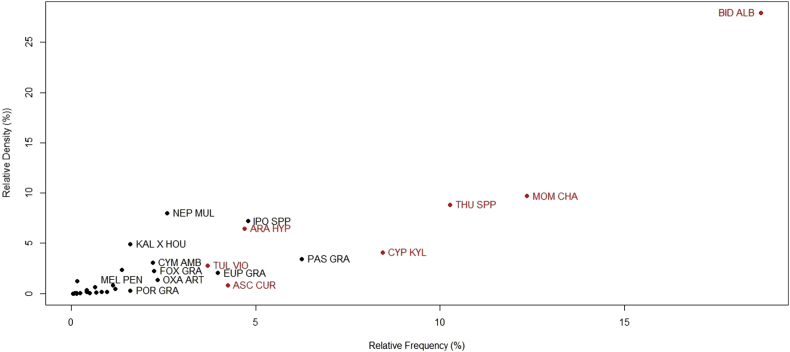
Relative density and frequency of species surveyed at the green roofs. Species in red are the most frequent, dense, or both. *Asclepias curassavica*, also in red, was highlighted because of its relevance to other projects held at the International Institute of Tropical Forestry.

We evaluated overall species frequency, density and importance, and results showed a set of species best suited to the tropical environmental conditions of the green roofs (Figure 5). *Bidens alba* was the only surveyed species found in all roof depths, and in almost all ranking first in IV. Species like, *Asclepias curassavica*, *Cyperaceae kyllinga*, *Momordica charantia*, *Oxalis articulata*, *Paspalum paniculatum*, and *Thunbergia spp* were also found in more than one roof depth, each in different frequencies and densities. *Asclepias curassavica* was not part of the originally planted set of species but used as part of an Institute project to attract monarch butterflies (*Danaus plexippus*).

On the other hand, a group of species were only found in specific roof depths, these species varied widely in terms of their relative dominance and frequency in each specific depth. *Cymbopogon ambiguous*, *Kalanchoes x houghtonii* had high importance values in the roof they were surveyed, but it is important to highlight they both were found on GR # 4 for which the depth is unknown. Also, even though *Alopecurus pratensis* was found in two different roofs, both have the same depth (10.16 cm), therefore we classified this species as depth specific. Other species were also found in only one roof depth but were not as dominant as the previous three (Table 3).

**Table 3:** Surveyed species Importance Value ranked by dominance. Highlighted species are depth specific and only appear in roofs with the same substratedepth.

SPECIES CODE	SPECIES NAME	GR # 1			GR # 2	GR # 3				GR # 4
		5.08 cm	7.62 cm	10.16 cm	10.16 cm	12.7 cm	15.24 cm	20.32 cm	25.4 cm	Unk. depth
BID ALB	*Bidens alba*	156.78	132.43	148.89	131.93	124.45	71.00	68.45	48.82	19.11
MOM CHA	*Momordica charantia*	0.00	58.62	15.14	6.56	118.47	118.27	102.92	97.39	0.00
THU SPP	*Thunbergia spp*	0.00	0.00	5.26	0.00	102.70	122.06	125.90	80.91	0.00
CYP KYL	*Cyperaceae kyllinga*	59.86	34.35	4.83	1.68	80.16	90.95	29.38	0.00	29.26
PAS GRA	*Paspalum paniculatum*	4.82	14.85	14.64	24.15	94.87	49.84	45.11	0.00	0.00
IPO SPP	*Ipomea spp*	0.00	0.00	0.00	0.00	49.39	0.00	150.83	31.25	0.00
ARA HYP	*Arachis hypogaea*	0.00	102.65	53.65	65.43	0.00	0.00	0.00	0.00	0.00
NEP MUL	*Nephrolepis multiflora*	61.70	72.58	0.00	27.96	0.00	0.00	0.00	0.00	0.00
EUP GRA	*Euphorbia graminea*	0.00	0.00	0.00	0.00	40.07	14.98	29.27	72.17	0.00
ASC CUR	*Asclepias curassavica*	4.82	9.60	39.95	4.90	58.97	31.66	0.00	4.92	0.00
TUL VIO	*Tulbaghia violacea*	61.93	25.84	47.89	18.03	0.00	0.00	0.00	0.00	0.00
CYM AMB	*Cymbopogon ambiguus*	0.00	0.00	0.00	0.00	0.00	0.00	0.00	0.00	104.49
KAL X HOU	*Kalanchoes x houghtonii*	0.00	0.00	0.00	0.00	0.00	0.00	0.00	0.00	99.71
FOX GRA	*Alopecurus pratensis*	0.00	0.00	96.42	1.61	0.00	0.00	0.00	0.00	0.00
OXA ART	*Oxalis articulata*	0.00	9.65	9.81	0.00	0.00	44.10	19.48	10.00	0.00
MEL PEN	*Melothria pendula*	0.00	0.00	0.00	0.00	0.00	0.00	0.00	68.66	0.00
POR GRA	*Portulaca grandiflora*	19.72	20.34	15.07	3.24	0.00	0.00	0.00	0.00	0.00
UNK 4-1	*Unknown species # 1*	0.00	0.00	0.00	0.00	0.00	0.00	0.00	0.00	46.15
PAS FOE	*Passiflora foetida*	0.00	0.00	0.00	35.47	0.00	0.00	4.82	4.92	0.00
POR OLE	*Portulaca oleracea*	0.00	0.00	35.05	0.00	0.00	0.00	0.00	0.00	0.00
EMI FOS	*Emilia fosbergii*	4.82	0.00	15.00	0.00	10.36	0.00	0.00	0.00	0.00
BUL CAU	*Bulbine caulescens*	6.44	0.00	0.00	21.71	0.00	0.00	0.00	0.00	0.00
OXA COR	*Oxalis corniculata*	0.00	0.00	0.00	10.40	14.51	0.00	0.00	0.00	0.00
SPE VER	*Spermacoce verticilata*	0.00	0.00	0.00	0.00	24.72	0.00	0.00	0.00	0.00
MAC LAT	*Macroptilium lathyroides*	0.00	0.00	0.00	18.01	0.00	0.00	0.00	0.00	0.00
PUR FLO	*Stachytarpheta jamaicensis*	0.00	0.00	17.36	0.00	0.00	0.00	0.00	0.00	0.00
KAL PIN	*Kalanche pinnata*	0.00	0.00	0.00	0.00	0.00	0.00	0.00	0.00	16.89
SPI ANT	*Spigelia anthelmia*	0.00	0.00	0.00	0.00	0.00	0.00	0.00	0.00	15.94
EMI SON	*Emilia sonchifolia*	0.00	0.00	4.83	0.00	0.00	0.00	0.00	0.00	3.49
DES SPP	*Desmodium spp*	0.00	0.00	0.00	0.00	5.07	0.00	0.00	0.00	0.00
TAL PAN	*Talinum paniculatum*	0.00	0.00	0.00	0.00	4.84	0.00	0.00	0.00	0.00
CIS VER	*Cissus verticilata*	0.00	0.00	0.00	0.00	0.00	0.00	4.82	0.00	0.00
SED STA	*Sedum Stahlii*	0.00	4.80	0.00	0.00	0.00	0.00	0.00	0.00	0.00
UNK 4-2	*Unknown species # 2*	0.00	0.00	0.00	0.00	0.00	0.00	0.00	0.00	3.75
POR PIL	*Portulaca pilosa*	0.00	0.00	0.00	3.50	0.00	0.00	0.00	0.00	0.00
DIO SPP	*Diodia spp*	0.00	0.00	0.00	0.00	0.00	0.00	0.00	0.00	2.33
POR AFR	*Portulacaria afra*	0.00	0.00	0.00	1.61	0.00	0.00	0.00	0.00	0.00

There were five common species, the rest were very widespread and/or sporadically located. *Bidens alba* was the most dense and frequent among all species regardless of the depth. Other species were frequent but not as dense, such as *Tulbaghia violacea* (IV: 61.9) and *Cyperaceae kyllinga* (IV: 59.8). GR # 2, with a substrate depth of 10.16 cm, showed a similar pattern as the first sampled roof in terms of the community composition; *Bidens alba* was the most dominant species (IV: 131.9); *Arachis hypogaea* was the second in IV (65.4), the third most important species was one that did not appear before, *Passiflora foetida*; this species was present in 2 of the three roofs sampled at the Institute, but more abundant in the GR # 2. In this roof there were two species that were only present within this area, e.g., *Macroptilium lathyroides* (IV: 18.1) and *Portulacaria afra* (IV: 1.6).

Green roof # 3, as described before is also subdivided in different substrate depths (12.7, 15.24, 20.32, and 25.4 cm) but the dynamics were very different for each of the sections and there was not a clear trend of dominance in this roof. The first subdivision with 12.7cm of substrate had a composition similar to the roofs sampled before. *Bidens alba* remaining the most dominant species (IV: 124.25). *Momordica charantia* was also common in this subdivision, ranking second (IV: 118.47). This species, even when present in the first two GRs, was not as dense or frequent. A set of species specific to this section were *Spermacoce verticilata* and *Talinum paniculatum*. At a depth of 15.24 cm a different species showed dominance over the *Bidens alba*. *Thunbergia spp* ranked first in Importance Value (122.06), and *Momordica charantia* remained in second place for this subdivision, (IV: 118.27). There were no species specific to this section and the composition was like the surrounding areas.

Similarly, on the 20.32 cm deep green roof section there were not many site-specific species, only *Cissus verticilata* (IV: 4.82). In this section what was more abundant was *Ipomea spp*, (IV: 150.83); this species was present only in this roof but in three out of four of its sections. The last section, and the deepest among all green roofs (25.4cm), contained species already sampled in previous roofs and sections, with the exception of the *Melothria pendula* (IV: 68.66), only surveyed within this depth. The dominant species of this section was the *Momordica charantia* (IV: 97.39).

The fourth sampled GR, as explained in the Methods section, not only is located at the Social Sciences Faculty at UPR-RP but it has different features; this influenced its vegetation composition. This roof contained some of the common species of previous green roofs, i.e., *Cyperacea kyllinga* (IV: 29.26), *Bidens alba* (IV: 19.11), and *Emilia sonchifolia* (IV: 3.49) but at different proportions. It also contained some new species such as *Cymbopogon ambiguus* (IV: 104.49) which was the most dominant, *Kalanchoes x hoightonii* (IV: 99.71) second most abundant and from the same family of the originally planted species.

## 4 Discussion

**Figure 6:**
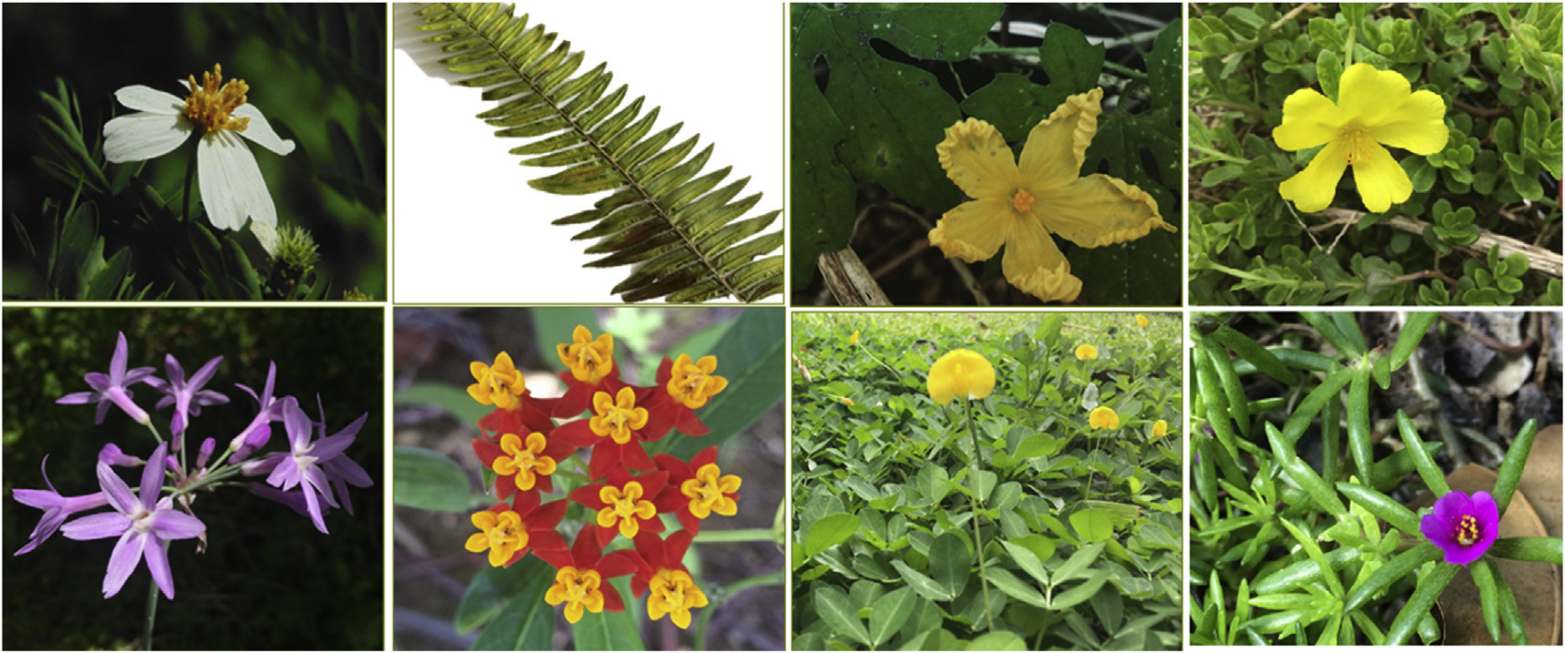
Some of the most common sampled species at the green roofs. From left to right, (on top): *Bidens alba*, *Nephrolepis multiflora*, *Momordica charantia*, *Portulaca oleracea*, (on the bottom): *Tulbaghia violacea*, *Asclepias curassavica*, *Arachis hypogaea*, *Portulaca pilosa* (Image by Author).

From our results we determined that vegetation included in the original design was mostly absent by the time we surveyed the sites (4 years after installment). It appears that many of the originally planted species were not necessarily suitable for the tropical roof top environment without constant maintenance, and, therefore, did not persist. Species like *Tulbaghia violacea*, *Asclepias curassavica* (a native planted later), and *Arachis hypogaea*, were the only species that seemed to be well adapted and persisted in the green roofs with high relative densities and/or frequencies (Figure 6). Most of the other species were not found at all, but some isolated individuals were found for species like *Passiflora foetida*, *Talinum paniculatum* and *Sedum stahlii*. We found scattered individuals of *Portulaca grandiflora*, *Portulaca oleracea* and *Portulaca pilosa* on some roofs. These are non-native succulent species, recommended for green roof design in tropical wet and dry conditions as stated by Vijayaraghavan (2016), so we suspect they may have been part of the originally planted ones. On the other hand, native species were well adapted and spread all over the green roofs almost regardless of the depth. The species that did outstandingly well were *Bidens alba*, *Nephrolepis multiflora*, and *Momordica charantia*; which were found at the highest importance values in more than one roof depth.

As previously mentioned, occasional trees were found on the green roofs (e.g. *Leucaena* spp. and *Spathodea* spp.), this aspect may correspond to the location of the GR in the botanical garden, but it is important to highlight that the presence of trees may affect other species development under them, this without maintenance or intervention (Hwang and Yue, 2015). Moreover, the presence of woody vegetation affects green roof durability and stability; therefore, a decision must be made surrounding the presence of this kind of species in green roofs with substrates not suited for their support on longer periods of time and as the root system develops, as they may affect community interactions and suppress diversity enhancement. Based on visual estimates, woody vegetation coverage occupied around 10–15% of the total roof area (all roofs included).

From these preliminary results we can propose a list of species that showed good adaptation to the local conditions, such as *Bidens alba*, *Tulbaghia violacea*, *Nephrolepis multiflora*, *Arachis hypogaea*, *Momordica charantia*, *Asclepias curassavica*, *Alopecurus pratensis*, *Paspalum paniculatum*, *Euphorbia graminea*, *Cymbopogon ambiguus*, and *Kalanchoes x houghtonii*. These are a mix of both native and non-native species that withstand not only the physical conditions but the competition of other species and the probable presence of local pests. All this ruderal species showed high or moderate relative densities and/or frequencies, which can be interpreted as good adaptation to the environment on the tropical green roofs or high resilience of the species to the conditions in these habitats. This species might provide a suitable mix to be included in future green roof designs in Puerto Rico as well as other tropical areas elsewhere.

As noted in the literature review, few considerations have been given to which species would be suitable for green roofs in the wet tropics. The region that has the best experience is Singapore and they have not only implemented good policies towards the implementation of green roofs but are pioneers in the tropical zone in the establishment of locally adapted species into their designs (Peng Lihua, 2012). Singapore has numerous studies that point out the benefits of this human-made ecosystems can be achieved in the tropics as well as in temperate zones, with the considerations of certain criteria, e.g., precipitation patterns. Even when precipitation and humidity are high in many tropical sites, water availability for plants could be limiting in some green roofs due to relatively shallow substrates and poorly design retention layers. Tan and Sia (2008) highlight some features that could be contemplated for plant selection; one of them is their photosynthesis mode. They suggest plants with Crassulacean Acid Metabolism (CAM) photosynthesis, because they are efficient in water use which is a great feature for dry areas or shallow green roofs. The National Parks Board of Singapore has been performing studies to delimit the plant list for green roof incorporation. Some of the species listed are *Alternanthera ficoidea*, *Bryophylhim fedtschenko*, *Carissa macrocarpa*, *Desmodium triflorum*, *Echeveria spp*., *Habranthus gracilifolius*, *Lobelia ehinensis*, *Plectranthus verticillatus*, *Wollastonia biflora*, among some others (Tan and Sia, 2008). These species might also be considered for green roofs in Puerto Rico in addition to the ones identified in this study. Green roof purpose can also narrow the species selection process, given GR are identified by different names and serve different purposes. One of the latest incorporations to GR utilities is the combination of its ecological features and agriculture, utilizing these spaces for the growing of edible species, decreasing the carbon footprint and increasing urban green infrastructure functionality.

Vegetation performance depends strongly on the conditions of the environment and the resource availability (Raimondo et al., 2015), especially since green roofs are engineered ecosystems that try to emulate ground level ones throughout the incorporation of artificial layers that need to be carefully balanced to retain the desire moisture and nutrients for the plants they hold. Green roofs are considered hostile environments for numerous species and the list of those that could adapt and survive high temperatures, dry conditions, and space limitations is scarce (Williams et al., 2014; Oberndorfer et al., 2007, Oberndorfer et al., 2007). Moreover, the substrate depth seems to be one of the key features that determines plant diversity and performance among green roofs as well as humidity retention. Extensive green roofs have an even smaller pool of options as potential plants for their design than intensive ones and, since the maintenance is often minimum, retaining the desired conditions is delegated to the moisture retention layer and substrate composition. If maintenance is regular the number of species could increment, and the environment can be artificially managed to comprise a different than natural plant composition. An option proposed to accomplish high survival rates and good propagation is the design from a functional diversity perspective (Van Mechelen et al., 2015, Van Mechelen et al., 2015b), this approach consists in allocating ecological traits among the green roofs in order to achieve the ecosystem resilience rather that aiming for a big list of species that would not survive on long-term and are not necessarily interconnected, these are the so called trade-offs of the ecosystem that could be involve in the design and species selection. Economic constraints are an important offset to these proposed approaches, most species selection is based on market availability and price and has little or nothing to do with ecological functions and services.

## 5 Conclusion

We recommend, the list of available species for green roof design needs to be evaluated from local condition adaptability and not from temperate climate previously selected lists, and that the purpose of the green roof needs to be well established to design aiming for better outcomes, as well as further development of tropical green roof adaptability studies. The surveyed sites need to be revisited to be able to establish if the results obtained in this study were from random conditions or if there is an actual trend towards local vegetation to take over species with low adaptability. Also, a decision from the owners of green roofs needs to be taken to establish management and maintenance treatments to maintain current conditions, to reverse to previous ones or to guide vegetation colonization towards a new composition and function from the green roofs.

Puerto Rico still needs further development in green roof research and design to enrich and promote the establishment and durability of this novel ecosystems in the Island. An inventory of private and public green roofs could be performed throughout the use of remote sensing tools, as well as an analysis of the public policy in the country towards sustainability strategies such as the green roof implementation incentives to assess optimum allocation, design durability and benefits. Also, for the sites used in this study, along with the vegetation surveys, water and energy efficiency analysis could be incorporated in future research.

## Declarations

### Author contribution statement

Iana Grullón-Penkova: Conceived and designed the experiments; Performed the experiments; Analyzed and interpreted the data; Wrote the paper.

Jess K. Zimmerman: Conceived and designed the experiments; Contributed reagents, materials, analysis tools or data.

Grizelle González: Contributed reagents, materials, analysis tools or data.

### Funding statement

This work was supported by the Luquillo Long-Term Ecological Research Site, 10.13039/100000001National Science Foundation [grant number DEB-1239764].

### Competing interest statement

The authors declare no conflict of interest.

### Additional information

No additional information is available for this paper.
